# Decentralized Assessment of a Dihydromyricetin- and L-Cysteine-Based Multi-Ingredient Dietary Supplement on Post-Alcohol Recovery: An Observational Cohort Study

**DOI:** 10.3390/nu18152424

**Published:** 2026-07-24

**Authors:** Sujin Song, Yan Wang, Lucas Atwood, Nirav R. Shah, Reza A. Jarral

**Affiliations:** 1 Clinical Excellence Research Center, Stanford University, Stanford, CA 94303, USA; susong@stanford.edu (S.S.); yanw914@stanford.edu (Y.W.); rjarral@stanford.edu (R.A.J.); 2 Atwood-Sciences, Denver, CO 81658, USA; lucasatwood@atwood-sciences.com; 3 CareHQ, Auckland 1010, New Zealand

**Keywords:** linear mixed-effects model, observational cohort, next-day functioning, dietary supplement, alcohol recovery, digital health

## Abstract

**Background/Objectives**: This decentralized, real-world observational cohort study assessed whether Cheers Restore (Houston, TX, USA), a commercially available dietary supplement, is associated with improved next-day subjective well-being (energy, mental clarity, physical well-being, and sleep quality) following alcohol consumption in healthy adults. A secondary aim evaluated the feasibility of integrating consumer wearables into future controlled studies. **Methods**: Ninety adults (56% female; mean age 44.4 years) were enrolled via the Alethios digital research platform and contributed 2958 morning surveys across three self-selected behavioral conditions: Alcohol-Only (reference), Cheers Restore, and Non-Drinking Baseline. Four subjective outcomes (energy, mental clarity, physical well-being, sleep quality rating; 1–10 scale) were modeled using linear mixed-effects models with random intercepts for participant and centered drink count as a covariate. **Results**: Cheers Restore use was associated with statistically significant improvements across all four subjective outcomes relative to alcohol-only nights: mental clarity (β = 0.356, 95% CI 0.214–0.499, *p* < 0.001), physical well-being (β = 0.335, 95% CI 0.189–0.482, *p* < 0.001), energy (β = 0.270, 95% CI 0.133–0.407, *p* < 0.001), and sleep quality rating (β = 0.263, 95% CI 0.096–0.430, *p* = 0.002). Standardized effect sizes were small (Cohen’s d = 0.20–0.32). Paired Alcohol Use Disorders Identification Test–Consumption (AUDIT-C) analysis confirmed stable consumption with no evidence of increased alcohol consumption over the short, self-reported follow-up. **Conclusions**: In this observational cohort, Cheers Restore use was associated with consistent improvements in next-day subjective well-being. These findings warrant a randomized, placebo-controlled trial to establish causality. ClinicalTrials.gov: NCT07069608, registered 16 July 2025.

## 1. Introduction

### 1.1. Background

Alcohol consumption is a prevalent modifiable lifestyle behavior with well-documented acute effects on sleep architecture, autonomic nervous system function, and next-day cognitive and physical performance [1]. These transient disruptions are of increasing interest to consumer health, where mild to moderate drinkers seek strategies to mitigate the negative impact of occasional, recreational alcohol consumption.

Dietary supplements containing amino acids, antioxidants, electrolytes, and plant-based compounds have been marketed for post-alcohol recovery, yet the evidence base remains limited [2]. Most existing studies rely on controlled laboratory paradigms with standardized alcohol challenges, which offer internal validity but sacrifice ecological relevance.

Decentralized digital research platforms enable large-scale, longitudinal monitoring of recovery in naturalistic settings. When combined with participant-reported outcome measures (PROMs) captured via mobile platforms, these data offer an ecologically valid approach to assessing lifestyle interventions.

### 1.2. Physiology

Post-alcohol recovery is a constellation of mental and physical symptoms that emerge as blood alcohol concentration (BAC) declines toward zero following a bout of drinking [3,4]. Although precise physiology remains incompletely understood, converging evidence implicates several interrelated mechanisms: direct toxicity of acetaldehyde, oxidative stress, immune dysregulation, neurochemical rebound, and micronutrient depletion [2,4,5].

Approximately 90% of ingested ethanol is metabolized oxidatively in the liver through two principal enzymatic pathways [3]. Alcohol dehydrogenase (ADH) catalyzes the conversion of ethanol to acetaldehyde, which is subsequently oxidized to acetate by mitochondrial aldehyde dehydrogenase (ALDH). A secondary pathway involving cytochrome P450 2E1 (CYP2E1) becomes quantitatively important during heavy consumption and generates substantial reactive oxygen species (ROS) as by-products [3,6]. The resulting shift in the hepatic NAD+/NADH ratio, together with CYP2E1-derived ROS, promotes lipid peroxidation, mitochondrial dysfunction, and inflammatory signaling [6]. Acetaldehyde itself is directly cytotoxic and depletes hepatic glutathione (GSH) stores, further compromising antioxidant defenses [7,8].

Beyond hepatic effects, acute ethanol exposure activates innate immune pathways through gut-barrier disruption and endotoxin translocation, elevating circulating pro-inflammatory cytokines such as TNF-α, IL-6, and IL-10 [5,9]. Neurochemically, ethanol potentiates γ-aminobutyric acid type A (GABAA) receptor activity during intoxication; the subsequent compensatory down-regulation may produce a rebound state that underlies many next-day symptoms [10,11]. Additionally, ethanol acutely inhibits intestinal absorption of B-group vitamins, vitamin C, vitamin E, and essential minerals, which may create transient micronutrient deficits that may exacerbate next-day effects [12,13].

Accordingly, proposed pharmacological and nutritional strategies for mitigating next-day effects target one or more of these mechanisms: accelerating acetaldehyde clearance via ADH/ALDH up-regulation, replenishing GSH and quenching ROS through antioxidant supplementation, modulating GABAergic rebound, and restoring depleted micronutrients [2,6,14].

### 1.3. Evidence Base for Recovery Supplement Ingredients

The recovery supplement (Cheers Restore) evaluated in the present study comprises ten active ingredients, each selected on the basis of preclinical or clinical evidence for activity along one or more of the pathways described above. Table 1 summarizes the putative mechanisms and key supporting references for each ingredient. The two principal bioactives, dihydromyricetin (DHM) and L-cysteine, are discussed in greater detail below, followed by a brief overview of the supporting micronutrients and botanical extracts.

#### 1.3.1. Dihydromyricetin (DHM)

Dihydromyricetin (also known as ampelopsin) is a polyphenolic flavonoid extracted from Ampelopsis grossedentata with a long history of use in traditional Chinese medicine for alcohol-related ailments [10,15]. In rodent models, DHM induces expression of ethanol-metabolizing enzymes ADH1, ALDH2, and ALDH1A1, thereby accelerating circulating ethanol and acetaldehyde clearance [15,16]. DHM also activates the AMPK pathway, attenuating ethanol-induced hepatic steatosis and improving mitochondrial lipid oxidation [17]. DHM stimulation of the Nrf2 antioxidant pathway suppresses CYP2E1-derived ROS [15]. Neurologically, DHM acts as a positive allosteric modulator at benzodiazepine sites on GABAA receptors in the hippocampus, counteracting the rebound hyperexcitability that follows ethanol withdrawal [10]. In a randomized, double-blind, placebo-controlled, crossover trial of 26 healthy Korean males with heterozygous ALDH2, a single dose of Hovenia dulcis fruit extract (rich in DHM) significantly reduced IL-6, IL-10, and AST levels and improved subjective next-day scores at 4 and 12 h post-administration [11].

#### 1.3.2. L-Cysteine

A semi-essential amino acid, L-Cysteine is a fundamental precursor for GSH, though research suggests ethanol metabolism disrupts the rate-limiting enzymatic steps of this synthesis [7]. By replenishing the hepatic GSH pool, exogenous L-cysteine directly counteracts the acetaldehyde-mediated GSH depletion that impairs phase-II detoxification [8,18]. When combined with DHM, enhanced enzymatic clearance (via ADH/ALDH up-regulation) and direct chemical neutralization of acetaldehyde represent a potentially synergistic strategy [15].

#### 1.3.3. Supporting Micronutrients and Botanical Extracts

The formulation additionally includes B-group vitamins (B1, B6, B12). Intestinal absorption of B-vitamins is acutely impaired by ethanol, and their supplementation preserves circulating B-vitamin levels during alcohol exposure [12,13]. Milk thistle (Silybum marianum), standardized to 80% silymarin, has established hepatoprotective and anti-inflammatory properties, acting primarily through inhibition of NF-κB and activation of the Nrf2 pathway [19,20]. Prickly pear (Opuntia ficus-indica) fruit has been shown to restore GSH levels and reduce erythrocyte damage in ethanol-exposed rats [21], with parallel reductions in oxidative-stress markers in healthy humans compared with vitamin C [22]. Ginger (Zingiber officinale) extract improved hepatic antioxidant status in ethanol-treated rats [23] with comparable MDA reduction to vitamin E in a drug-induced liver injury model [24], and has been shown to protect against ethanol-induced renal damage in rats [25]. Vitamin C reduced ethanol-induced radical formation and prevented endothelial dysfunction [26], while vitamin E attenuated alcohol-induced oxidative stress and restored hepatic redox status in mice [27]. Table 1 provides a consolidated summary of each ingredient, dosage, primary mechanism of action, and key supporting references.

**Table 1:** Active ingredients in Cheers Restore, putative mechanisms of action relevant for next-day recovery, and key supporting evidence. Evidence type (human, animal, or in vitro) is indicated within the Key Evidence column; where a species is not stated, the cited findings derive from preclinical (rodent or cell-based) models.

Ingredient *	Dose per Serving	Primary Mechanism(s) *	Key Evidence *
Dihydromyricetin (DHM)	1200 mg	Up-regulates ADH/ALDH expression; activates AMPK/SIRT1/PGC-1α pathway; Nrf2-mediated antioxidant induction; GABAA receptor modulation	Reduced hepatic steatosis and inflammation in mouse ethanol-feeding models [10,15,16,17]; decreased AST, IL-6, IL-10, and subjective recovery scores in human crossover trial (*n* = 26) [11]
L-Cysteine HCl	450 mg	GSH precursor; direct acetaldehyde scavenging via MTCA formation; redox homeostasis maintenance	Chronic ethanol depletes hepatic cysteine in antioxidant pathways [7]; cysteine derivatives prevent ethanol-induced GSH depletion in animal models [8]; cysteine + glutathione reduces acetaldehyde and hepatic injury in mice [18]
Vitamin C (ascorbic acid)	75 mg	Water-soluble free-radical scavenger; increases NO bioavailability; regenerates α-tocopherol	Reduced ethanol-induced radical formation; prevented endothelial dysfunction [26] (animal/ex vivo)
Vitamin E (dl-α-tocopheryl acetate)	40 mg	Lipid-phase peroxyl radical scavenger; protects membrane integrity from ethanol-induced lipid peroxidation	Supplementation in mice significantly prevented alcohol-induced oxidative stress and apoptosis in the liver while restoring redox status [27]
Thiamin (B1)	20 mg	Cofactor in alcohol metabolism (TPP); antioxidant properties; supports neural function	Ethanol may inhibit B1 absorption; supplementation preserves B-vitamin levels during ethanol exposure [12] (human review)
Vitamin B6 (pyridoxine)	20 mg	Coenzyme in >100 reactions in protein and energy metabolism; hepatic release enhanced by ethanol	Ethanol impairs B6 absorption, distribution, and excretion; supplementation offsets depletion [12,13]
Vitamin B12 (cyanocobalamin)	100 µg	Cofactor in methionine synthesis and mitochondrial energy metabolism	Ethanol disrupts B12 absorption; supplemental B-vitamins preserve circulating levels in rat models [12,13]
Milk Thistle (*S. marianum*)	50 mg	Silymarin: NF-κB inhibition, Nrf2 activation, ROS scavenging, iron/copper chelation	Meta-analysis of 5 RCTs (*n* = 1198) showed reduced drug-induced liver injury; hepatoprotective in multiple models [19,20,28,29]
Prickly Pear (*O. ficus-indica*)	50 mg	Polyphenol- and betalain-mediated GSH restoration; anti-inflammatory cytokine modulation	Restored GSH and reduced erythrocyte damage in ethanol-exposed rats; decreased MDA in humans vs. vitamin C [21,22,30]
Ginger (*Z. officinale*)	50 mg	Gingerol/shogaol-mediated COX-1 inhibition; antioxidant enzyme activation; gastric motility support	Improved hepatic and renal antioxidant status in ethanol-treated rats [23,25]; comparable to vitamin E in reducing MDA in a drug-induced liver injury model [24]; supportive ginger antioxidant review [31]

* **Abbreviations**: ADH, alcohol dehydrogenase; ALDH, aldehyde dehydrogenase; AMPK, AMP-activated protein kinase; CYP2E1, cytochrome P450 2E1; GABAA, γ-aminobutyric acid type A; GSH, glutathione; MDA, malondialdehyde; MTCA, 2-methylthiazolidine-4-carboxylic acid; NF-κB, nuclear factor kappa B; NO, nitric oxide; Nrf2, nuclear factor erythroid 2-related factor 2; PGC-1α, peroxisome proliferator-activated receptor gamma coactivator 1-alpha; ROS, reactive oxygen species; SIRT1, sirtuin 1; TPP, thiamine pyrophosphate.

### 1.4. Objectives

Primary Aim: To assess whether Cheers Restore use is associated with improved next-day subjective well-being (energy, mental clarity, physical well-being, and sleep quality rating) following alcohol consumption, relative to alcohol-only nights.

Secondary Aim: To characterize the dose–response relationship between alcohol quantity and recovery outcomes; to compare all drinking-night conditions against a non-drinking baseline; and to quantify within- and between-person variability to inform future randomized controlled trial design.

Hypothesis: Use of Cheers Restore following alcohol consumption is associated with improved next-day self-reported functioning compared to alcohol-only nights.

## 2. Materials and Methods

### 2.1. Study Design

This was a decentralized, 6-week, prospective observational cohort study conducted between June 2025 and January 2026 using the Alethios™ (San Francisco, CA, USA) digital research platform (Protocol 1002, v.1.2; ClinicalTrials.gov: NCT07069608, registered 16 July 2025). The study employed a within-subject repeated-measures design in which each participant contributed nightly observations across three behaviorally defined conditions using morning surveys: (1) Alcohol-Only nights (alcohol consumption without Cheers Restore), (2) Cheers Restore nights (alcohol consumption followed by Cheers Restore use), and (3) Non-Drinking Baseline nights (no alcohol consumption). No interventions were assigned; participants self-selected their behavior each night and logged it retrospectively via the platform. This report follows the Strengthening the Reporting of Observational Studies in Epidemiology (STROBE) guidelines for cohort studies [32].

### 2.2. Setting

All study procedures were conducted remotely. There were no in-person visits. Participants were recruited nationally and completed all data collection via the Alethios web-based application, which facilitated electronic informed consent, daily behavioral logging, morning well-being surveys, weekly WHO-5 well-being assessments, and passive wearable device synchronization. The study was approved by Sterling IRB and conducted in compliance with HIPAA, 21 CFR Part 11, and applicable ethical guidelines. All participants provided electronic informed consent prior to data collection.

### 2.3. Participants

#### 2.3.1. Eligibility Criteria

Eligible participants were adults aged ≥ 21 years who consumed alcohol on ≥1 night per week, owned a compatible wearable device (Fitbit, Apple Watch, Garmin, Whoop, or Oura Ring), and were willing to log alcohol consumption, supplement use, and daily well-being via the Alethios platform. Exclusion criteria included AUDIT-C scores above gender-specific hazardous drinking thresholds (≥4 for men, ≥3 for women), history of alcohol use disorder or substance misuse treatment, routine binge drinking (≥5 drinks/occasion for men, ≥4 for women), use of THC, recreational drugs, or prescription stimulants, sedatives, or sleep aids, diagnosis of sleep or psychiatric conditions (insomnia, sleep apnea, major depressive disorder, PTSD, bipolar disorder, schizophrenia, ADHD), pregnancy or breastfeeding, and inability to comply with platform-based data collection.

#### 2.3.2. Enrollment and Follow-Up

A total of 111 participants were enrolled via the Alethios platform. Of these, 20 were excluded for not initiating daily morning surveys, yielding 91 participants with AM survey records (the demographic sample). One further participant was excluded for insufficient data (22 vs. 41 expected records), producing a primary analysis sample of 90 participants. All remaining 90 participants met the pre-specified threshold of ≥80% individual survey adherence and were retained for primary analysis. Each participant was expected to contribute 15–25 usable nights of data. Survey completion adherence was 79.8% (2964 of 3712 possible survey-days), yielding 2958 analyzable survey-nights for subjective outcomes.

### 2.4. Variables

#### 2.4.1. Exposure

The primary exposure variable was a self-selected nightly behavioral condition, a three-level categorical variable: Alcohol-Only (reference), Cheers Restore, and Non-Drinking Baseline. Condition status was determined by participants and self-reported each morning. As a continuous covariate, the number of alcoholic drinks consumed was centered at the sample mean of 1.30 to allow the condition effects to be interpreted at the average drink count.

#### 2.4.2. Primary Outcomes

Four subjective morning well-being measures were identified (1–10 Likert scale): energy level, mental clarity, physical well-being, and sleep quality rating.

#### 2.4.3. Covariates

The primary LMEMs reported include alcohol quantity (centered) as the principal covariate. Exploratory models initially included the following covariates for inclusion in adjusted models: alcohol quantity (continuous), beverage type, and day of week. However, beverage type introduced multicollinearity with condition and drink count, and the model exhibited rank deficiency, automatically dropping one or more beverage categories. Most associated coefficients were not statistically significant. Day of week similarly failed to improve model fit. These terms were therefore excluded from the primary models, retaining only centered drink count as the covariate most directly relevant to the primary research question.

### 2.5. Data Sources and Measurement

#### 2.5.1. Wearable Data Collection

Wearable data were collected passively from participants’ personal consumer-grade devices (Apple Watch, Fitbit, Garmin, Oura Ring, and Whoop) synchronized through the Alethios platform. Device type was not controlled, and participants used their existing personal devices, which introduced measurement heterogeneity across manufacturers but reflects real-world usage patterns. Per-device adherence was tracked across the cohort to evaluate feasibility for future controlled studies; wearable data analyses are reserved for future work and are not reported in this manuscript.

#### 2.5.2. Self-Reported Outcomes

Morning well-being surveys were administered daily via the Alethios mobile application. Participants rated their energy, mental clarity, physical well-being, and sleep quality on a 1–10 integer scale upon waking. Alcohol consumption (number and type of drinks) and Cheers Restore usage were logged concurrently. Supplement dose and timing of intake were self-reported and not independently verified. Adverse events were monitored throughout the study via the platform’s participant event-reporting function, in accordance with the study protocol. The use of single-item, investigator-designed scales (1–10) for morning well-being outcomes was a pragmatic design choice to maximize daily compliance in a decentralized, real-world setting where participants completed surveys repeatedly over six weeks. Validated multi-item self-rated recovery instruments, while psychometrically stronger, impose greater respondent burden and risk attrition in naturalistic longitudinal designs. The consistency of the observed effects across all four self-report domains provides some reassurance regarding measurement sensitivity, though the absence of validated instruments limits direct comparison with prior clinical trials. A confirmatory randomized controlled trial should incorporate validated self-reported severity scales to strengthen construct validity and enable cross-study comparison.

### 2.6. Bias

Several design features were implemented to mitigate potential sources of bias. The within-subject design controls for time-invariant confounders (genetics, baseline physiology, personality traits) by using each participant as their own control. Centering of the drink count variable adjusts for the dose–response effect of alcohol quantity, reducing confounding by occasion-specific consumption levels. However, the non-randomized, self-selected nature of supplement use introduces the possibility of systematic differences between conditions (e.g., participants may preferentially use Cheers Restore on heavier drinking occasions or social events), which cannot be fully addressed without randomization. Additionally, morning self-report measures are subject to expectancy effects, due to participants being aware of having taken the supplement, who then may rate their well-being more favorably (open-label bias).

### 2.7. Study Size

The target enrollment of approximately 100 participants (90 completers) was determined by power simulations for repeated-measures mixed-effects models. With 15–25 nights per participant across conditions, the study was powered to detect small-to-moderate effect sizes (Cohen’s d = 0.20–0.32) in sleep and recovery metrics with >80% power at α = 0.05. The final analytic sample of 2958 survey nights from 90 participants exceeded minimum requirements for subjective outcomes.

### 2.8. Statistical Methods

#### 2.8.1. Primary Analysis

All primary outcomes were modeled using linear mixed-effects models (LMEMs) fitted via restricted maximum likelihood (REML) using the lme4 package (version 2.0-6) in R, with the lmerTest extension used to provide *p*-values via Satterthwaite’s degrees of freedom approximation [33,34]. Each outcome Y for participant i on night j was modeled as:

Y_ij_ = β_0_ + β_1_ · Condition_NDB, ij_ + β_2_ · Condition_Cheers, ij_ + β_3_·NumDrinks_ij_^centered^ + u_i_ + ε_ij_

where β_0_ is the intercept (mean outcome on Alcohol-Only nights at the mean drink count for an average participant), β_1_ and β_2_ are fixed effects for Non-Drinking Baseline and Cheers Restore conditions relative to Alcohol-Only, β_3_ is the fixed effect of centered drink count, u_i_ ~ N(0, σ^2^_u_) is the random intercept for participant i, and ε_ij_ ~ N(0, σ^2^_ε_) is the residual error. In lme4 syntax, the model was specified as:

lmer(Outcome ~ ConditionGroupF + NumOfDrinksUpdatedC + (1|ParticipantID), data = *df*, REML = TRUE)

The random intercept accounts for the non-independence of repeated observations within participants and captures stable between-person differences in outcome levels. Random slopes for condition were not included because of the unbalanced condition distribution (57.7% NDB, 26.1% Cheers Restore, 16.1% Alcohol-Only) and the modest number of observations per condition, within participants, produced convergence failures when random slopes were specified. The random-intercept-only model provided stable estimation and adequate fit across all eight outcomes.

#### 2.8.2. Variance Partitioning

Intraclass correlation coefficients (ICCs) were computed as ICC = σ^2^_u_/(σ^2^_u_ + σ^2^_ε_) to quantify the proportion of total outcome variance attributable to between-person versus within-person (night-to-night) differences.

#### 2.8.3. Effect Size Estimation

Standardized effect sizes were derived as the ratio of fixed-effect coefficients to the total standard deviation (combining between- and within-person variance components), providing a metric analogous to Cohen’s d for interpretation.

#### 2.8.4. Missing Data

Data completeness was monitored in real time via the Alethios platform. LMEMs accommodate unbalanced data under the assumption of missing at random (MAR), as they do not require equal observations per participant and use all available data for estimation without imputation.

## 3. Results

### 3.1. Participants

**Figure 1:**
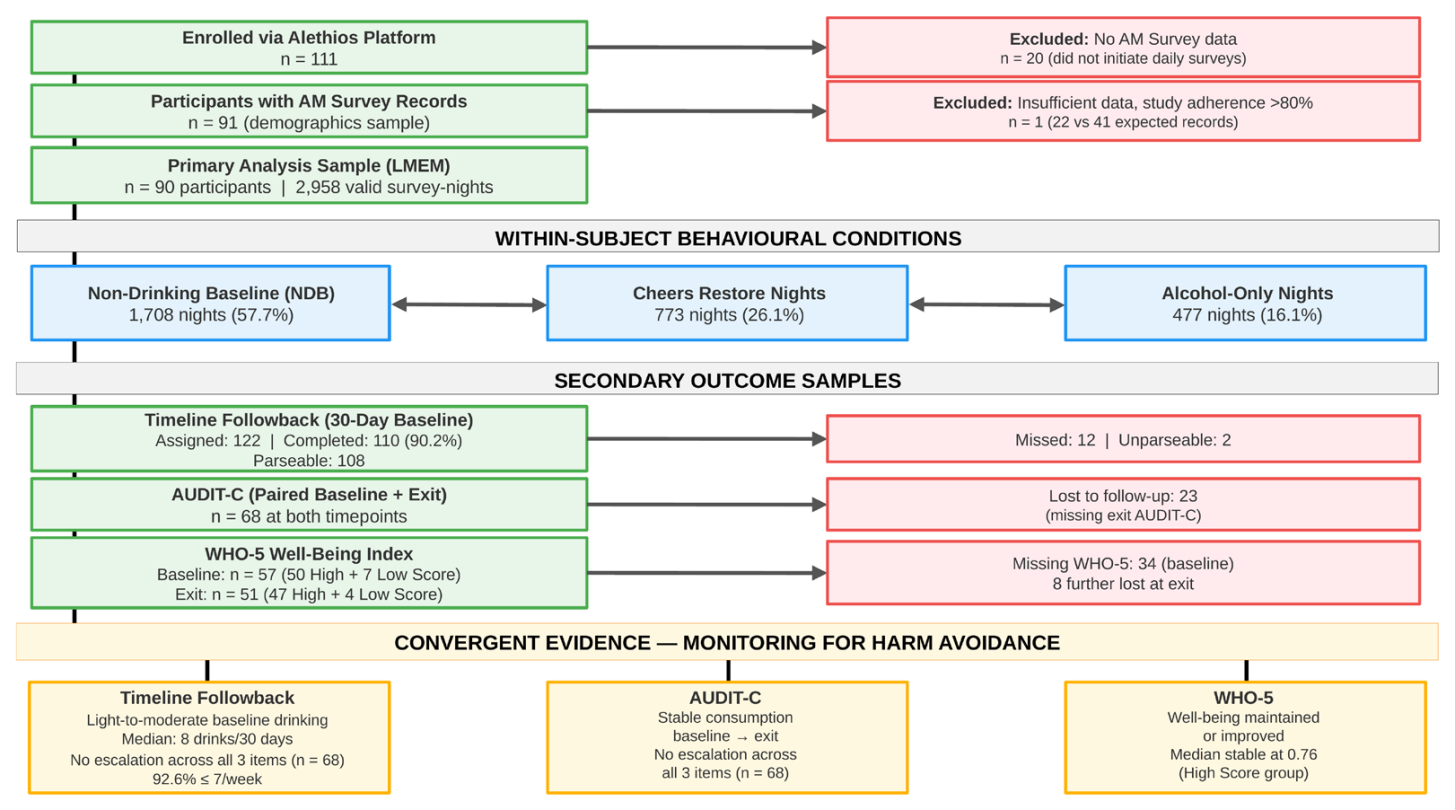
Participant flow diagram showing enrollment (*n* = 111), exclusions (*n* = 21), primary analysis sample (*n* = 90, 2958 survey-nights), within-subject behavioral condition distribution, and secondary outcome measures (TLFB, AUDIT-C, WHO-5).

Of 111 individuals enrolled via the Alethios platform, 20 were excluded for not initiating daily AM surveys and 1 for insufficient data (22 vs. 41 expected records), yielding a primary analysis sample of 90 participants contributing 2958 analyzable survey nights (approximately 33 nights per participant; Figure 1). Survey adherence was 79.8% (2964 of 3712 possible survey-days). Within the primary sample, behavioral condition distribution was: Non-Drinking Baseline 1708 nights (57.7%), Recovery Supplement 773 nights (26.1%), and Alcohol-Only 477 nights (16.1%).

### 3.2. Descriptive Data

Table 2 presents the demographic and behavioral characteristics of the study cohort.

### 3.3. Main Results

#### 3.3.1. Subjective Morning Well-Being Outcomes

**Figure 2:**
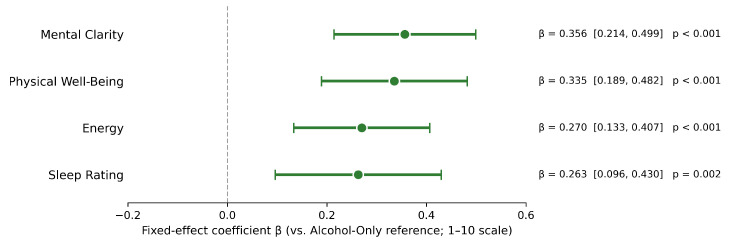
Forest plot of Recovery Supplement fixed-effect coefficients (β) with 95% confidence intervals from linear mixed-effects models. Reference condition = Alcohol-Only.

**Figure 3:**
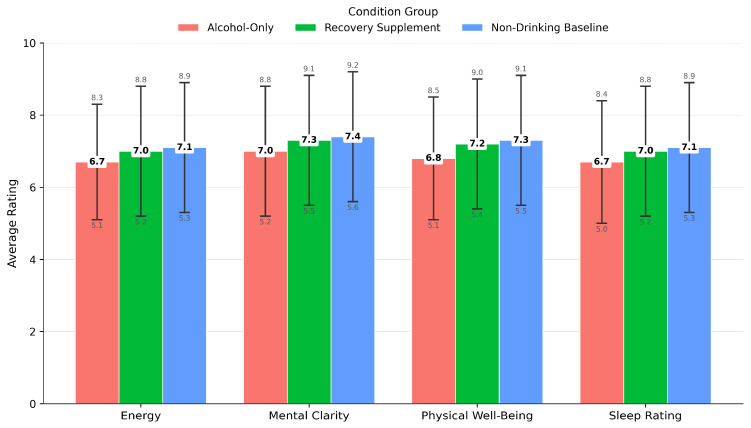
Mean descriptive survey ratings with error bars by condition group: Alcohol-Only, Recovery Supplement, and Non-Drinking Baseline.

All four subjective outcomes demonstrated statistically significant improvements for the Cheers Restore condition relative to Alcohol-Only (Table 3; Figure 2). The largest effect was observed for mental clarity (β = 0.356, 95% CI 0.214–0.499, *p* < 0.001, d = 0.315), followed by physical well-being (β = 0.335, 95% CI 0.189–0.482, *p* < 0.001, d = 0.288), energy (β = 0.270, 95% CI 0.133–0.407, *p* < 0.001, d = 0.249), and sleep quality rating (β = 0.263, 95% CI 0.096–0.430, *p* = 0.002, d = 0.198). In practical terms, the supplement closed 50–80% of the gap between alcohol-impaired and sober mornings across raw descriptive means (Figure 3): Physical Well-Being 80%, Energy 75%, Mental Clarity 75%, and Sleep Rating 50% (Cohen’s d = 0.17–0.23).

**Figure 4:**
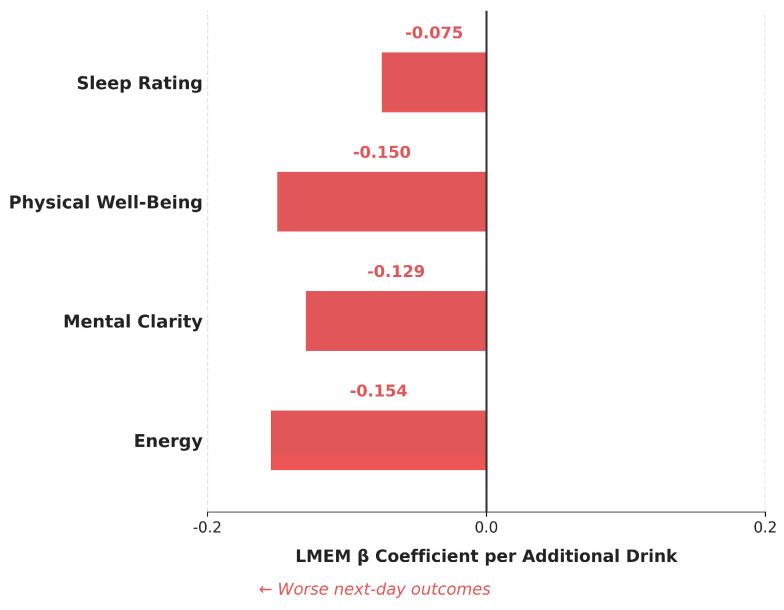
Dose–response coefficients from linear mixed-effects models showing the LMEM β coefficient for each additional alcoholic drink consumed. All subjective dose–response coefficients significant at *p* < 0.001.

The Non-Drinking Baseline condition was also significantly better than Alcohol-Only across all four subjective outcomes (all *p* < 0.05), confirming the expected negative impact of alcohol on next-day well-being. Sleep rating showed the largest NDB advantage (β = 0.408, *p* < 0.001). Alcohol quantity (centered) was a significant negative predictor across all four subjective outcomes (β range: −0.075 to −0.154, all *p* < 0.001). As shown in Figure 4, the estimated linear mixed-effects model coefficients were consistently negative for every outcome, demonstrating a robust dose–response relationship between increasing alcohol consumption and poorer next-day well-being.

#### 3.3.2. Secondary Outcomes: Drinking Patterns and Well-Being

Three secondary outcome measures provided convergent evidence regarding harm reduction and drinking pattern stability during the observation period.

Timeline Followback (TLFB). Of 122 TLFB assessments assigned, 110 were completed (90.2%) and 108 were parseable. The cohort reported a median of 8 drinks per 30 days, with 92.6% falling in the low-risk category (0–7 drinks per week). Period-level analyses showed stable consumption across four 7–9 day segments, with a mean of 0.39 drinks per day (2.72 drinks per week).

AUDIT-C (Paired Baseline and Exit). Among 68 participants with paired baseline and exit AUDIT-C data (23 lost to follow-up at exit), scores remained stable across all three items (drinking frequency, typical quantity, heavy episodic drinking), with no evidence of supplement-facilitated escalation in alcohol consumption.

WHO-5 Well-Being Index. At baseline, 57 participants completed the WHO-5 (50 in the High Score group, 7 in the Low Score group). At exit, 51 participants remained (47 High, 4 Low). The High Score group maintained a stable median of 0.76 at both timepoints. The Low Score group showed improvement from a median of 0.36 to 0.46, though the small subgroup size (*n* = 4–7) limits interpretation. Given the very small low-baseline subgroup, this apparent improvement should be regarded as exploratory only.

Taken together, these secondary measures indicate that Recovery Supplement use operated within existing drinking patterns and was not associated with increased alcohol consumption or diminished general well-being during the 6-week observation period.

### 3.4. Variance Components and Model Diagnostics

**Figure 5:**
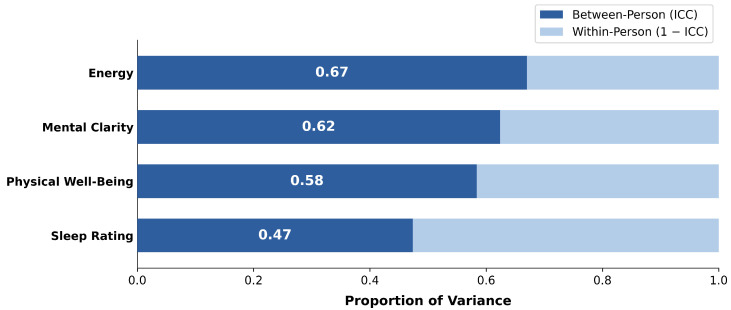
Intraclass correlation coefficients (ICCs) showing the proportion of total variance attributable to between-person versus within-person differences for each outcome.

**Figure 6:**
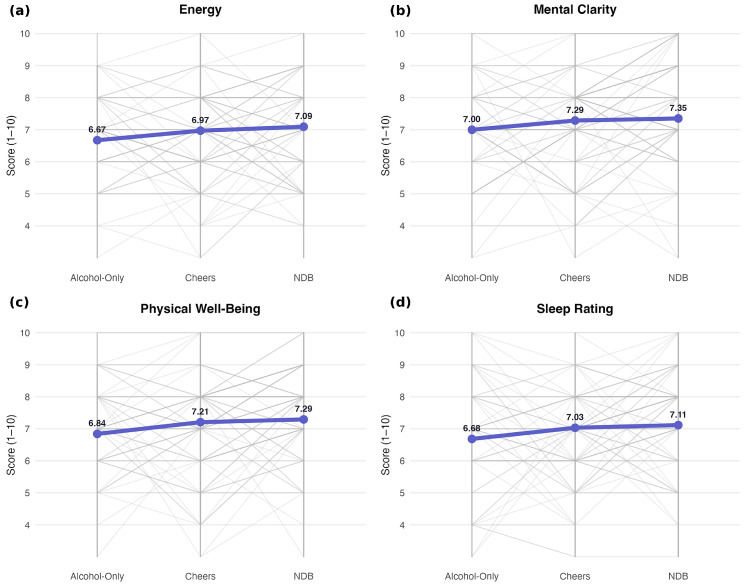
Individual participant trajectories across behavioral conditions. (**a**) Energy ratings; (**b**) Mental clarity ratings; (**c**) Physical well-being ratings; (**d**) Sleep quality ratings. Faint gray lines represent individual participant trajectories (*n* = 90), illustrating substantial between-person variability, while the bold indigo line shows the group mean for each outcome across Alcohol-Only, Recovery Supplement, and Non-Drinking Baseline conditions.

ICCs for the four subjective outcomes ranged from 0.474 (sleep rating) to 0.670 (energy), reflecting a balanced distribution of between- and within-person variance (Figure 5). Individual participant trajectories across conditions are shown in Figure 6, illustrating both the consistent direction of supplement-associated benefit and the substantial between-person variability characteristic of real-world observational data.

## 4. Discussion

### 4.1. Key Results

This study examined whether Cheers Restore, a dietary supplement containing antioxidants, electrolytes, and plant-based compounds, was associated with improved next-day recovery outcomes following alcohol consumption in a real-world observational setting. Using linear mixed-effects models applied to 2958 survey nights from 90 participants, we found that Cheers Restore use was associated with statistically significant improvements in all four subjective well-being measures, after adjusting for alcohol quantity and between-person variability. Effect sizes were small (Cohen’s d = 0.20–0.32) but consistent across outcomes. These findings support the study’s primary hypothesis with respect to subjective well-being outcomes.

### 4.2. Interpretation

The observed subjective improvements are consistent with the broader literature on post-alcohol recovery supplements, which has reported modest benefits for symptoms such as headache, fatigue, and cognitive fog through mechanisms involving antioxidant support, electrolyte repletion, and hepatoprotective compounds [2]. The effect magnitudes in this study (β ≈ 0.27–0.36 on a 10-point scale) are comparable to those reported in laboratory-based alcohol challenge studies evaluating similar formulations, though direct comparison is limited by differences in study design, dosing, and outcome measures. Although all four outcomes reached statistical significance, the standardized effect sizes were small (Cohen’s d = 0.20–0.32), and no established minimal clinically important difference exists for these single-item scales, so the clinical relevance of improvements of this magnitude remains uncertain. Their consistency across all four domains and the observed dose–response gradient are supportive but do not by themselves establish a clinically meaningful benefit. Systematic reviews of hangover interventions have similarly concluded that the overall evidence base remains limited and of low methodological quality [35], reinforcing that these associations require confirmation in randomized, placebo-controlled trials.

A related interpretive note concerns the relative magnitude of the Non-Drinking Baseline (NDB) and Cheers Restore β coefficients. For several subjective outcomes, the NDB coefficient is smaller than the Cheers Restore coefficient (e.g., Energy: NDB β = 0.149 vs. Cheers β = 0.270), which might appear to suggest that supplement use confers greater benefit than abstaining from alcohol entirely. This is an artifact of the model’s covariate structure rather than a substantive finding. Because the LMEM includes centered drink count as a covariate, condition-level β coefficients are estimated at the mean drink count. On NDB nights, actual drink count is zero, well below the sample mean, so the benefit of not drinking is partially absorbed by the drink-count coefficient rather than the condition coefficient. The Cheers Restore β, by contrast, is estimated from nights where participants consumed alcohol at or near the average level, producing a more direct comparison against Alcohol-Only nights at comparable consumption. Raw descriptive means in Figure 3 confirm that NDB nights yield the highest absolute well-being scores across all four outcomes.

### 4.3. Strengths

This study leverages several methodological strengths. The decentralized design provides ecologically valid data on real-world behavior patterns, capturing naturalistic alcohol consumption and recovery rather than laboratory-standardized conditions. The within-subject repeated-measures framework, analyzed via LMEMs, accounts for the hierarchical data structure and controls for stable individual differences, a critical advantage given the high ICCs observed for many outcomes. The large number of total observations (2958 survey nights) provides robust statistical power for subjective endpoints, and the inclusion of centered drink count as a covariate adjusts for the dose–response confound inherent in observational alcohol research. The Alethios platform’s real-time monitoring ensured high survey adherence (79.8%), and the study was conducted under IRB oversight with HIPAA-compliant data handling.

A deliberate design choice was the use of an observational cohort with a bring-your-own-device (BYOD) wearable protocol as a precursor to a controlled trial. This approach served to evaluate the feasibility of integrating consumer-grade wearables into future controlled studies.

### 4.4. Limitations

#### 4.4.1. Observational Design and Self-Selection Bias

Participants self-selected when to use Cheers Restore, introducing the possibility of confounding by indication. Individuals may use the supplement preferentially on nights of heavier drinking, social occasions, or when anticipating the need for recovery. These conditions may systematically differ from alcohol-only nights in ways not captured by drink count alone. Without randomization, causal inference is not possible. Because Cheers Restore is a fixed, multi-ingredient commercial product whose use was self-reported rather than verified, its effects cannot be attributed to any single constituent.

#### 4.4.2. Open-Label Expectancy Bias

Participants were aware of their supplement use, which may inflate subjective ratings through placebo or expectancy effects. Open-label placebo effects can persist even when participants are explicitly informed they are receiving a placebo, indicating that expectancy can operate independently of conscious belief in efficacy [36,37]. In addition, the four subjective outcomes were assessed with single-item, investigator-designed scales rather than validated instruments, limiting psychometric comparability with prior clinical trials. Future confirmatory trials should therefore employ validated recovery or hangover-severity instruments to enable psychometric benchmarking and direct comparison with prior clinical trials.

#### 4.4.3. Limited Covariate Adjustment

The primary models adjust only for drink count. Additional covariates (e.g., beverage type, day of the week) were explored but were excluded due to multicollinearity, rank deficiency, or lack of improvement in the model fit. Consequently, residual confounding remains possible. In particular, several time-varying factors that were not captured by the platform, including food intake, hydration, sleep duration, physical activity, caffeine and nicotine use, concomitant medications, and the social or situational drinking context, may influence next-day well-being independently of supplement use. Together with the self-selected timing of supplement use (confounding by indication), this residual confounding means the observed associations should be interpreted as exploratory rather than causal. Formal sensitivity analyses and additional model-fit statistics, such as marginal and conditional R^2^, are planned for the confirmatory randomized trial.

#### 4.4.4. Industry Sponsorship

The study was funded by the manufacturer of the evaluated supplement. Although the analyses were conducted independently by the academic investigators and the sponsor did not alter the study design, delivery, or analytical conclusions, industry funding can introduce potential bias, and independent replication will be important to confirm these findings.

### 4.5. Generalizability

The enrollment process screened a substantial number of candidates, many who were excluded based on drinking behavior criteria. This selective enrollment raises questions about the representativeness of the sample relative to the broader population of individuals who consume alcohol. The enrolled study cohort was predominantly low-risk drinkers (92% low-risk AUDIT-C), well-educated (63% bachelor’s/master’s), and technologically engaged adults. This is representative of the target consumer market for recovery supplements but may not generalize to populations with different drinking patterns, socioeconomic profiles, or technology access. The exclusion of individuals with hazardous drinking, sleep disorders, or psychiatric conditions limits applicability to clinical populations. Results should be interpreted as applicable to healthy, moderate adult drinkers using the supplement in a naturalistic context. Future controlled trials may consider broadening inclusion criteria while implementing appropriate safety monitoring, including real-time adverse event reporting and Data Safety Monitoring Board oversight. The cohort was also predominantly White (79.1%), further limiting demographic representativeness. In addition, individuals who elect to use a recovery supplement may adopt other health-related recovery behaviors that were not captured here (healthy-user bias), which could contribute to the observed associations.

### 4.6. Future Research

The present findings provide a robust observational foundation and critical design parameters for a confirmatory randomized controlled trial. Based on the observed effect sizes and variance components, we recommend a single device, randomized, double-blind, placebo-controlled crossover trial with the following parameters: a composite primary endpoint with validated questionnaires [38,39], *n* = 250 enrolled participants (targeting 200 completers), a minimum 7-day washout period between crossover arms, and a standardized single wearable device type to reduce physiological measurement noise. Power calculations based on the observed composite effect size (d ≈ 0.27) indicate that 116 participants in a crossover design would provide 90% power at α = 0.05. As performed for this study, future research will be pre-registered on ClinicalTrials.gov with CONSORT-compliant reporting. Pre-specified exploratory analyses in that trial should examine responder versus non-responder profiles, sex- and age-related differences in response, and genetic moderators of alcohol metabolism such as ALDH2 polymorphisms.

## 5. Conclusions

In this real-world, decentralized observational cohort, use of Cheers Restore was associated with consistent, statistically significant improvements in next-day subjective well-being following alcohol consumption. These findings support further investigation via a randomized, placebo-controlled trial to establish causal efficacy and quantify the role of expectancy effects. Because the study was observational and open-label, these findings should be regarded as exploratory and hypothesis-generating: causal inference will require a randomized, placebo-controlled trial.

## Figures and Tables

**Table 2:** Participant characteristics (*n* = 91, demographic sample).

Characteristic	Value	Detail
**Demographics**		
Age, mean (SD, range)	44.4 years	SD 11.2; range 22–74
20–29	7.7%	*n* = 7
30–39	29.7%	*n* = 27
40–49	30.8%	*n* = 28
50–59	22.0%	*n* = 20
60+	9.9%	*n* = 9
**Sex**		
Female	56.0%	*n* = 51
Male	44.0%	*n* = 40
**Race/Ethnicity**		
White	79.1%	*n* = 72
Black or African American	8.8%	*n* = 8
Asian	6.6%	*n* = 6
Hispanic or Latino	2.2%	*n* = 2
Native American/Alaska Native	2.2%	*n* = 2
Multi-racial	1.1%	*n* = 1
**Marital Status**		
Married	52.7%	*n* = 48
Single	20.9%	*n* = 19
Divorced	9.9%	*n* = 9
Living with partner	9.9%	*n* = 9
Other	6.6%	*n* = 6
**Education**		
Bachelor’s degree	31.9%	*n* = 29
Master’s degree	30.8%	*n* = 28
Doctorate/Professional degree	9.9%	*n* = 9
Some graduate school	7.7%	*n* = 7
Associate degree	5.5%	*n* = 5
Some college/High school/GED	14.3%	*n* = 13
**Employment**		
Full-time employed	65.9%	*n* = 60
Part-time employed	9.9%	*n* = 9
Retired	7.7%	*n* = 7
Homemaker	5.5%	*n* = 5
Self-employed	4.4%	*n* = 4
Other	6.6%	*n* = 6
**Beverage Preference (Drinking Nights)**		
Beer	26.7%	of total selections
Wine/Champagne	24.2%	of total selections
Mixed (multiple types)	27.1%	of total selections
Spirits	12.7%	of total selections
Cocktail/Mixed Drink	6.5%	of total selections
Hard Seltzer/Other	2.7%	of total selections
**Drinking Risk (AUDIT-C, All Drinking Nights)**		
Low Risk (≤4 drinks)	91.9%	*n* = 2349 nights
Moderate Drinking (5–7 drinks)	6.2%	*n* = 159 nights
Heavy Drinking (8+ drinks)	1.8%	*n* = 47 nights
**Study Participation**		
Survey Adherence	79.8%	2964/3712 survey-days
Total Analyzable Survey-Nights	2958	Subjective outcomes

**Table 3:** Linear mixed-effects model results: fixed-effect estimates by outcome.

Outcome	*n* obs	*n* subj	Cheers β (95% CI)	*p*-Value	NDB β (95% CI)	Drinks β	ES (d)
Energy	2958	90	0.270 (0.133, 0.407)	<0.001 ***	0.149 (0.004, 0.293)	−0.154 ***	0.249
Mental Clarity	2958	90	0.356 (0.214, 0.499)	<0.001 ***	0.270 (0.119, 0.420)	−0.129 ***	0.315
Physical WB	2958	90	0.335 (0.189, 0.482)	<0.001 ***	0.215 (0.060, 0.370)	−0.150 ***	0.288
Sleep Rating	2958	90	0.263 (0.096, 0.430)	0.002 **	0.408 (0.232, 0.584)	−0.075 ***	0.198

Notes: Reference condition = Alcohol-Only. β = unstandardized regression coefficient from LMEM. ES (d) = standardized effect size for the Cheers condition. ** *p* < 0.01, *** *p* < 0.001. All models include random intercept for participant and centered drink count as covariate.

## Data Availability

De-identified participant-level data supporting the findings of this study may be made available upon reasonable request to the corresponding author, subject to IRB-approved data sharing agreements. This study was conducted on a proprietary platform owned by Alethios. All non-proprietary materials will be made available to readers.

## Abbreviations

The following abbreviations are used in this manuscript:

ADH	alcohol dehydrogenase
ADH1	alcohol dehydrogenase 1
ADHD	attention deficit hyperactivity disorder
AIC	Akaike information criterion
ALDH	aldehyde dehydrogenase
ALDH1A1	aldehyde dehydrogenase 1 family member A1
ALDH2	aldehyde dehydrogenase 2
AMPK	AMP-activated protein kinase
AO	alcohol-only
AST	aspartate aminotransferase
AUDIT-C	Alcohol Use Disorders Identification Test–Consumption
B1	thiamine
B12	cobalamin
B6	pyridoxine
BAC	blood alcohol concentration
BIC	Bayesian information criterion
bpm	beats per minute
CI	confidence interval
CONSORT	Consolidated Standards of Reporting Trials
COX-1	cyclooxygenase 1
CYP2E1	cytochrome P450 2E1
DHM	dihydromyricetin
ES	effect size
GABAA	γ-aminobutyric acid type A
GSH	glutathione
HIPAA	Health Insurance Portability and Accountability Act
ICC	intraclass correlation coefficient
IL-10	interleukin 10
IL-6	interleukin 6
IRB	institutional review board
LMEM	linear mixed-effects model
MAR	missing at random
MDA	malondialdehyde
MTCA	2-methylthiazolidine-4-carboxylic acid
NAD+	nicotinamide adenine dinucleotide
NADH	nicotinamide adenine dinucleotide reduced form
NDB	non-drinking baseline
NF-κB	nuclear factor kappa B
NN	normal-to-normal intervals
NO	nitric oxide
Nrf2	nuclear factor erythroid 2-related factor 2
PGC-1α	peroxisome proliferator-activated receptor gamma coactivator 1-alpha
PROMs	patient-reported outcome measures
PTSD	posttraumatic stress disorder
RCT	randomized controlled trial
REM	rapid eye movement
REML	restricted maximum likelihood
ROS	reactive oxygen species
SD	standard deviation
SIRT1	sirtuin 1
THC	tetrahydrocannabinol
TLFB	Timeline Followback
TNF-α	tumor necrosis factor alpha
TPP	thiamine pyrophosphate
WHO-5	World Health Organization Five Well-Being Index
21 CFR Part 11	Title 21 Code of Federal Regulations Part 11
